# Prophylactic low-dose hydrocortisone in neonates born extremely preterm: current knowledge and future challenges

**DOI:** 10.1038/s41390-024-03756-6

**Published:** 2024-11-26

**Authors:** Olivier Baud, Héloïse Torchin, Marine Butin, Cyril Flamant, Alexandra Nuytten

**Affiliations:** 1 Obstetrical Perinatal and Pediatric Epidemiology Research Team, EPOPE, French Institute for Medical Research and Health, Université Paris Cite, CRESS, INSERM, INRAE, Paris, France; 2 Department of Neonatal Medicine, Cochin Port-Royal Hospital, FHU PREMA, AP-HP Centre, Paris, France; 3 NeuroDiderot, Université Paris Cité, INSERM, Paris, France; 4 Department of Neonatology, Hospices Civils de Lyon, Hôpital Femme Mère Enfant, Bron, France; 5 Centre International de Recherche en Infectiologie, Equipe “Pathogénie des Infections à Staphylocoques”, INSERM U1111, CNRS UMR 5308, ENS de Lyon, Université Claude Bernard Lyon 1, Lyon, France; 6 Neonatal Intensive Care Unit, University Hospital of Nantes, Nantes, France; 7 Neonatal Intensive Care Unit, Saint Vincent de Paul Hospital, GHICL, Lille, France

## Abstract

**Summary:**

Prophylactic administration of low-dose hydrocortisone, at replacement dosage, targets inability of extremely low gestational age neonates (ELGANs) to respond to postnatal stress due to adrenal glands immaturity and is intended to prevent serious complications such as death and bronchopulmonary dysplasia (BPD). Increasing evidence from systematic reviews shows that prophylactic hydrocortisone reduces pre-discharge mortality, improves survival without BPD, favors patent ductus arteriosus (PDA) closure, and may have beneficial effects on cardiovascular stability and urine output. In contrast, an increased risk of spontaneous intestinal perforation when prophylactic hydrocortisone is combined with indomethacin and late-onset sepsis, particularly in infants of 24–25 weeks of gestation, have been reported as major adverse events. No significant negative impact on long-term neurodevelopmental outcomes following prophylactic hydrocortisone exposure was observed. Recent real-world data, despite their intrinsic methodological limitations, generally confirm the benefits observed in clinical trials, even with additional potential benefits and without increased adverse events. Ongoing challenges and questions discussed in this invited review relate to the best population to treat, optimal timing and duration of treatment, and potential barriers to implementation due to evolving knowledge and guidelines.

**Impact statement:**

- Prophylactic low-dose hydrocortisone improves survival without BPD in infants born extremely preterm.
- Recent real-world data generally confirm the benefits observed in clinical trials, even with additional potential benefits and without increased adverse events.
- Unanswered questions remain about optimal timing and duration of treatment, and potential barriers to implementation due to evolving knowledge and guidelines.

## Rationale for using prophylactic hydrocortisone in neonates born extremely preterm

**Fig. 1:**
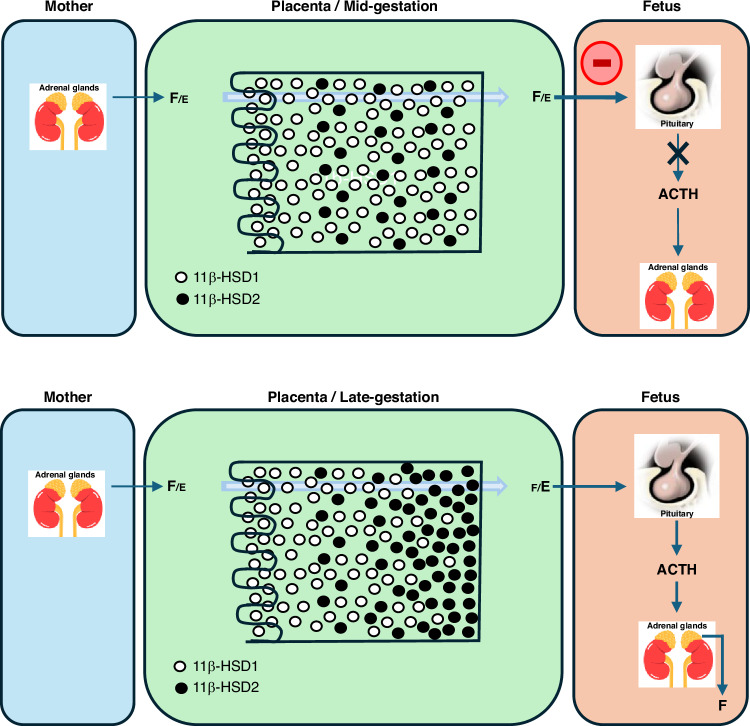
HSD11b1 and HSD11b2 expression in the baboon syncytiotrophoblast during gestation. Subcellular localization of HSD11b1 and HSD11b2 in the baboon syncytiotrophoblast, at mid-and late gestation. F: cortisol; E: cortisone. Adapted from ref.^58^

The strategy of using hydrocortisone early after birth in extremely low gestational age neonates (ELGANs) has its origins in the inability of the adrenal glands to respond to postnatal stress under difficult conditions.^1^ Indeed, the adrenal glands in ELGANs are particularly immature,^2^ which increases their vulnerability to many stressors commonly encountered in the neonatal intensive care unit, such as ventilator support and high oxygen levels, temperature fluctuations and metabolic instability, and pain associated with various invasive procedures. The tissue-specific distribution of 11β-HSD1 and 11β-HSD2 in the placenta and fetal membranes favors the conversion of maternal cortisol to cortisone as gestation progresses, both in baboons and in human amniotic fibroblasts^3^ (Fig. 1). While the inactivation of maternal cortisol to cortisone by placental 11β-HSD2 occurs in late gestation,^4^ the low expression of 11β-HSD2 in the placenta earlier in the gestation leads to an extra-adrenal source of glucocorticoids *in utero*, inducing a negative feedback of fetal HPA axis.^5^ Deprivation at birth of this maternal source of cortisol may partly explain the transient reduction in cortisol levels of the most immature infants. In addition, it has been suggested that the limited enzymatic capacity to synthesize cortisol is causally related to elevated cortisol precursors found in ELGANs.^6,7^ In a normal pregnancy, the transitional zone of the adrenal gland appears at 22–24 weeks gestation, and the expression of 3β-HSD then gradually increases until birth.^2^ However, extremely preterm labor is frequently due to chorioamnionitis reported to be associated with abnormal development of the adrenal glands and higher serum cortisol concentrations shortly after birth.^8^

Cortisol is crucial for various physiological processes, including lung maturation, maintenance of blood pressure and glucose metabolism. In premature infants, inadequate cortisol levels can lead to respiratory distress syndrome, hypotension, hypoglycemia, and other complications associated with prematurity, including in particular death and bronchopulmonary dysplasia (BPD).^9,10^

Both in neonatal baboons and humans, extremely preterm delivery was found to be associated with decreased cortisol production and hemodynamic failure reversed by hydrocortisone supplementation,^9–12^ and also shown to expedite weaning of vasopressor support in hemodynamically-compromised preterm infants.^13^

Several studies suggest a potential benefit of cortisol replacement in ELGANs, not limited to hemodynamic adaptation, but also to lung development and protection against persistent inflammation. Indeed, lower cortisol levels or a diminished cortisol response to ACTH have been associated with the development of BPD.^1^ In addition, various exogenous glucocorticoids may have different targets, in example reduced mortality rate for hydrocortisone and more inflammatory-related targets for dexamethasone.^14^ The prophylactic approach to preventing adrenal insufficiency was reconsidered in the early 2000s in response to the adverse neurodevelopmental effects reported following the use of dexamethasone, the main glucocorticoid used to prevent BPD at the time. While the beneficial effect of low-dose prophylactic hydrocortisone without neurodevelopmental impairment is now well accepted,^1,15^ it was associated with some adverse events potentially due to an interaction with concurrent treatments or clinical practices.^15^ The benefit-risk ratio is still unclear and this review will unravel some of the key unanswered questions regarding the prophylactic use of hydrocortisone in ELGANs.

## Prophylactic hydrocortisone and survival BPD: what is the true effect size?

BPD is a major complication of extreme prematurity and has few treatment options.^16,17^ Postnatal steroid use is still controversial,^18^ but prophylactic low-dose hydrocortisone has been found to prevent both the functional adrenal insufficiency that occurs in extreme prematurity and the deleterious effects of inflammation on the developing lung.^19^

The PREMILOC trial, using the lowest dosage reported so far (0.5 mg/kg q12h for 7 days from birth, followed by 0.5 mg/kg q24h for 3 additional days), showed a significant increase of 9% in BPD-free survival in extremely preterm infants exposed to prophylactic hydrocortisone (60% in the hydrocortisone group versus 51% in the placebo group, OR = 1.48 [95% CI, 1.02–2.16], *p* = 0.04), NNT = 12 (95% CI 6–200),^20^ notably without neurodevelopmental adverse outcomes.^21–23^ A recent meta-analysis based on individual patient data (IPD) was recently published and summarized in Table 1. It further confirmed the benefits of prophylactic hydrocortisone in improving not only survival without BPD (OR, 1.45; 95% CI, 1.11–1.90; *P* = 0.007; *I*^2^ = 0%) but also by reducing death before discharge (OR, 0.70; 95% CI, 0.51–0.97; *P* = 0.03; *I*^2^ = 0%), and reducing the need of medical treatment for patent ductus arteriosus (OR, 0.72; 95% CI, 0.56–0.93; *P* = 0.01; *I*^2^ = 0%).^24^ However, the PREMILOC trial excluded subpopulations such as ELGANs with severe intra-uterine growth restriction (IUGR) and preterm rupture of the membranes below 20 weeks, implying that treatment effect within these subgroups cannot be assessed. Neonates born following pregnancy complicated by chorioamnionitis showed better size effect of prophylactic hydrocortisone. Indeed, survival without BPD at 36 weeks (aOR, 2.01; 95% CI, 1.19–3.39; *P* = 0.009) and death before discharge (aOR, 0.43; 95% CI, 0.23–0.82; *P* = 0.010) were two outcomes strongly improved by the treatment in infants with chorioamnionitis compared to those without (aOR, 1.40; 95% CI, 0.97–2.02; *P* = 0.074; aOR, 0.71; 95% CI, 0.44–1.15; *P* = 0.16), when adjusted for sex, gestational age, and any antenatal steroids.

**Table 1:** Main benefits and risks of the individual patient data meta-analysis of neonates exposed to prophylactic hydrocortisone recruited in randomized controlled trials from 2001 and 2014 (adapted from Shaffer et al.).

Outcome	n/N (%)		aOR (95% CI)	*P*-value	*I*²	NNT/NNH	Comment
	Hydrocortisone	Placebo					
Benefits							
Survival without BPD	258/484 (53.3)	225/495 (45.5)	1.45 (1.11–1.90)	0.007	0%	13	NNT = 6 when adjusted to baseline characteristics.^25^
BPD at 36 weeks PMA	147/405 (36.3)	172/397 (43.3)	0.73 (0.54–0.98)	0.038	0%	14	BPD at 40 weeks PMA significantly prevented by early hydrocortisone as well.^32^
Death before discharge	85/485 (17.5)	112/497 (22.5)	0.70 (0.51–0.97)	0.033	0%	20	
Medical treatment for PDA	202/485 (41.7)	246/497 (49.5)	0.72 (0.56–0.93)	0.012	0%	13	
Risks							
Spontaneous intestinal perforation	35/483 (7.3)	15/497 (3.0)	2.50 (1.33–4.69)	0.004	32%	24	Observed neither in the PREMILOC trial nor in real-world data.^48–50^
Late onset sepsis	176/485 (36.3)	148/497 (29.8)	1.34 (1.02–1.75)	0.04	0%	15	Without any detectable negative impact on BPD, death or NDI.^24^

*aOR* Odds Ratios adjusted for sex, gestational age and antenatal steroids, *I*² summarizes the statistical heterogeneity, *NNT* number needed to treat, *NNH* number needed to harm, *BPD* Bronchopulonary dysplasia, *PDA* patent ductus arteriosus.

A recent re-analysis of the PREMILOC trial failed to find any heterogeneity in the treatment effect in specific subpopulations for which the benefit/risk ratio may be optimized.^25^ Furthermore, the effect of the drug proved to be relatively constant in the different centers. After adjusting for baseline covariates strongly predictive of survival without BPD, we found an odds ratio of 1.82 (95%CI 1.16–2.85, *p* = 0.009), which was higher than that originally reported (OR = 1.48, 95%CI 1.02–2.16, *p* = 0.04). This corresponds to a risk ratio of RR = 1.29 [1.07–1.48] or equivalently an absolute risk difference ARD = 14.6% [0.04–24.3], or the number needed to treat NNT = 6.85 [4.16–27]. These data are reassuring results regarding the magnitude and direction of the prophylactic hydrocortisone effect in all preterm subpopulations.

## Prophylactic hydrocortisone: is survival without BPD the only short-term benefit?

In addition to its primary goal, i.e., increasing BPD-free survival in ELGANs, prophylactic hydrocortisone has also been associated with several other beneficial effects. The most consistent is the spontaneous closure of the PDA, possibly caused by the anti-inflammatory effect of prophylactic hydrocortisone, which inhibits the release of arachidonic acid and prostaglandin synthesis. Indeed, in the IPD meta-analysis, a significant decrease in treatment of PDA was observed (OR, 0.72; 95%CI 0.56–0.93, *p* = 0.01, *I*² = 0%).^24^ In addition, in the PREMILOC trial, a significant reduction in surgical ligation was also reported.^20^ This effect is consistent with findings reported after prophylactic use of inhaled budesonide (NEUROSIS trial).^26^ and with the association between adrenal insufficiency and PDA-related complications.^27^ These findings are of special interest while recent data showed no clear benefit of early PDA closure with non-steroidal anti-inflammatory drugs.^28,29^ A recent meta-analysis and meta-regression involving 7 trials of prophylactic and selective hydrocortisone for a total of 2193 ELGANs showed a significant reduction in the incidence of necrotizing enterocolitis (RR: 0.72 (95%CI: 0.53–0.92), *p* < 0.001), a result that was unexpected and therefore should be taken with caution.^30^ Nevertheless, one explanation would be the positive treatment effect on blood pressure and PDA closure, which supports mesenteric perfusion, which is one of the players associated with the risk of necrotizing enterocolitis. The cardiovascular effects of hydrocortisone in preterm infants with hypotension lead to increased urine output reduced needs for dopamine, dobutamine, and/or epinephrine. Overall, hydrocortisone resulted in a rapid normalization of cardiovascular status and a sustained decrease in volume and pressor requirements.^31^ Recent data also provide evidence of the beneficial effect of prophylactic hydrocortisone on preventing BPD at 40 weeks of PMA.^32^ Finally, the potential beneficial effect of prophylactic hydrocortisone on the incidence of retinopathy of prematurity requiring treatment has recently been observed based on a real-world database but remains to be confirmed.

## Potential hydrocortisone-related adverse event: evidence of causality and prevention

The discussion on the postnatal use of corticosteroids cannot be separated from a thorough investigation of possible adverse effects. Regarding the early administration of prophylactic hydrocortisone in ELGANs, three potential adverse effects deserve special attention.

First, an increased risk of spontaneous intestinal perforation (SIP) in neonates receiving prophylactic hydrocortisone, the main reason why two randomized controlled trials conducted in the 2000s were discontinued.^33,34^ Authors suggested a possible cumulative effect with early indomethacin, known for its risk of gastrointestinal perforation.^35^ The more recent PREMILOC trial did not report an increased incidence of SIP associated with early low-dose hydrocortisone because the study protocol prohibited the use of ibuprofen in the first 24 h of life.^20^ While the IPD meta-analysis confirmed the association between this treatment and the risk of SIP,^15^ the adjusted subgroup analysis highlights a significant association with indomethacin (aOR = 9.37 [2.02–43.49]), which was not observed in the absence of indomethacin (aOR = 1.52 [0.73–3.15]). These results emphasize the need for caution in the concomitant use of non-steroidal anti-inflammatory drugs and prophylactic hydrocortisone in ELGANs. Finally, recent real-world data suggest that there is no additional risk following the introduction of prophylactic hydrocortisone in several countries including the UK, Canada, Sweden, and the US. Furthermore, an ancillary study to the PREMILOC trial suggests that elevated baseline cortisol levels in infants exposed to prophylactic hydrocortisone may be associated with an increased risk of SIP (aOR 4.81 [1.34–17.22]) without additional improvement of BPD-free survival.^36^ These findings are exploratory only but consistent with the physiologic rationale for the use of prophylactic hydrocortisone as replacement therapy in ELGANs, which is no longer indicated when endogenous cortisol levels are high after birth.

An increased risk of late-onset sepsis was also associated with prophylactic hydrocortisone treatment, particularly in the subgroup of infants born at 24–25 weeks of gestation (OR = 1.87 CI 95% [1.09–3.21]),^20^ a finding confirmed in the IPD meta-analysis (OR 1.34 [1.02–1.75], *p* = 0.04). However, this potential adverse event did not result in an additional risk for neonatal mortality, mortality at discharge or neurodevelopmental disorders at 2 or 5 years of age. Because most events were recorded distant from prophylactic hydrocortisone exposure and the incidence of late-onset sepsis varied widely between centers, the basis for this effect remains unclear. It should also be noted that no increased incidence of BPD or abnormal neurodevelopmental outcomes was associated with this excess rate of late-onset sepsis. Prospective cohort studies of this adverse event are currently being conducted in the USA and Sweden to assess this potential risk in current practice.

Finally, an important potential side effect that should be avoided by using prophylactic hydrocortisone instead of dexamethasone is neurodevelopmental disorders. Most of the available data are reassuring in this respect. In all randomized trials examining the administration of prophylactic hydrocortisone in ELGANs, no neurodevelopmental impairments were found, and even with some weak evidence of benefits.^21,23,24^ The MRI examinations of the brain were also not significantly altered by the hydrocortisone exposure, which also had no significant effect on the gross anatomical findings,^37^ white matter microstructure, and microstructural connectivity in major cerebral and cerebellar pathways.^38^

Interestingly, these long-term outcomes are fully consistent with the lack of adverse neurodevelopmental effects reported in recent large randomized clinical trials using selective hydrocortisone at much higher cumulative doses within the first 3 weeks of life.^39–41^

## How to identify the best target population to improve benefit/risk ratio of prophylactic hydrocortisone treatment?

One of the biggest challenges in any prophylactic intervention is to identify the most relevant population at birth to avoid unnecessary exposure to potentially unaffected infants. Two main strategies have recently emerged with the use of predictive models and the identification of infants with proven adrenal insufficiency.

In the PREMILOC trial, 50% of recruited ELGANs survived without BPD in the placebo group. While the potential benefit of prophylactic hydrocortisone for children who will not develop BPD may be limited, it could be much greater for children at high risk of BPD. The main BPD risk factors available at birth are gestational age (BPD at 36 weeks PMA occurs in 50–80% of infants born at 24 weeks of gestation and in 10–30% of those born at 27 weeks),^42^ sex, birth weight, and especially fetal growth restriction,^43^ and severe respiratory distress. Other perinatal events that are strongly associated with progression of BPD, such as necrotizing enterocolitis or late-onset sepsis, cannot be considered when deciding on prophylactic treatment because they occur later.

The challenge is to individualize prophylactic hydrocortisone treatment for infants at high risk of BPD. For many decades, several scores have been developed to accurately identify ELGANs at risk of death or BPD. However, a recent systematic review concluded that although the models for predicting BPD perform satisfactorily, they all have a high risk of bias. Therefore, methodological improvements are needed before they can be considered for use in clinical practice.^44^ We have recently developed a new prediction model that includes variables that are only available at or shortly after birth. It is based on the literature and the Neonatal Research Network BPD estimator.^45^ We were able to improve the discriminatory power of the latter by adding candidate variables at birth associated with BPD. The modified model with the best predictive power included gestational age at birth, birth weight, respiratory support at birth, sex, center effect, and multiple pregnancy as predictors at birth (auROC [95% CI] = 0.85 [0.82–0.88]). Whichever prediction model is used, you need to know how well it performs in the target population in terms of discrimination (C-index), calibration (graphical methods), accuracy (proportion of well-classified children), and net benefit.^46^ Finally, the use of a prediction model also implies the choice of a threshold value for the predicted risk above which prophylactic hydrocortisone treatment is carried out. In the medical community, there is no uniform threshold. However, some data may guide clinical practice:

- according to the meta-regression published by Doyle et al. showed that postnatal steroids significantly increased the chance of death or cerebral palsy when risk for BPD remains below 30%, whereas it reduced this chance with risk for BPD over 70%,^47^
- success in treatment infants with prophylactic hydrocortisone was reported better when a priori probability to survive without BPD at baseline ranged from 25 to 50%.^25^

Identification of infants with proven adrenal insufficiency can be based on baseline cortisol measurements at or shortly after birth. In a prespecified secondary analysis of the PREMILOC trial, we found no predictive value of baseline cortisol serum concentration for BPD-free survival in ELGANs treated with hydrocortisone, but a serum cortisol concentration above 889 nmol/L (32 µg/dL) was associated with a higher risk of severe intraventricular hemorrhage and in treated infants.^36^ Therefore, high cortisol levels early after birth may reduce the benefit/risk ratio for prophylactic hydrocortisone treatment in ELGANs.

## Prophylactic hydrocortisone use in real world

Since the publication of the PREMILOC trial and the IPD meta-analysis, many NICUs have introduced prophylactic hydrocortisone treatment as a standard of care in ELGANs and reported on their experiences. Despite some limitations, mainly related to the historical study design, these reports are of interest to better assess the impact and safety of prophylactic hydrocortisone in routine care. Clinical trials, which are considered the gold standard for assessing the benefits and risks of medical interventions, evaluate the safety and efficacy of investigational drugs according to predefined (not always pragmatic) protocols in limited (and sometimes selected) patient samples. In contrast, real-world data refers to data derived from various non-experimental or observational sources that reflect routine clinical practice. Evidence obtained from the analysis of real-world data, despite their intrinsic limitations, provides complementary information on use, efficacy, and safety in broader patient populations under real-world conditions.

To our knowledge, the first real-world report came from a single-center retrospective cohort in UK, in which outcomes of ELGANs were compared before and after prophylactic hydrocortisone implementation.^48^ In comparison to the pre-implementation period (control group, *n* = 103), the incidence of SIP in the hydrocortisone group (*n* = 88) was 7.7% (vs 3.4% *p* = 0.2), NEC 30.0% (vs 25.0% *p* = 0.43) and LOS 34.0% (vs 30.6% *p* = 0.63). Rates of BPD-free survival was of 26.2% (vs 27.2% *p* = 0.87). Infants who received prophylactic hydrocortisone had a significantly lower requirement of inotropic support of 32.0% vs 48.3% (*p* = 0.02). Authors concluded that prophylactic hydrocortisone was not associated in significant increase in serious adverse effects in ELGANs in their cohort.

In a Canadian single tertiary NICU,^49^ 76 and 71 ELGANs were compared before and after using prophylactic hydrocortisone without significant adverse effects but a potential benefit on discharge without O_2_ and on high-grade intraventricular hemorrhage.

In USA, a cohort of very sick ELGANs including some infants below 24 weeks of gestation was reported.^50^ After risk adjustment, hydrocortisone-treated infants demonstrated improved BPD-free survival and did not increase rates of SIP or sepsis.

In preliminary reports, two Swedish NICUs reported an effect size of prophylactic hydrocortisone treatment comparable to the main results already reported in both RCTs and meta-analyses, with no adverse events, in particular no increased risk of SIP and late-onset sepsis. A possible effect of prophylactic hydrocortisone on the incidence of retinopathy of prematurity requiring treatment remains to be further analyzed due to conflicting data.

Pending further data, prophylactic hydrocortisone use in the real world appears to have very similar effects on survival without BPD compared to controlled trials, perhaps with additional benefits. More importantly, these benefits do not appear to be attenuated by the two main adverse events, i.e., SIP and sepsis, reported in trials completed 10–25 years ago, suggesting that advances in neonatal care of ELGANs may have positively altered the safety profile of this treatment in the interim.

## Ongoing challenges and barriers

While there are quite solid conclusions about the benefits of prophylactic hydrocortisone and its safety for the neurological development of infants born extremely preterm, many questions remain. Some of these relate directly to the differences between dexamethasone and hydrocortisone. While dexamethasone is characterized by a longer half-life and a lack of mineralocorticoid action, hydrocortisone is thought to bind mineralocorticoid receptors at low doses and glucocorticoid receptors at higher doses, thus acting more like an endogenous cortisol-like molecule. These differences may explain additional effects of prophylactic hydrocortisone in other developing systems including PDA closure and safety on the developing brain,^51,52^ while it potentially produces less effect than dexamethasone for reducing lung inflammation and disruption in lung development.^53^

Other questions are related to the main indication for treatment. In addition to the selection of populations eligible for adrenal dysfunction with cortisol replacement, as discussed above, there is also the question of the gestational age threshold for systematic treatment. Treating all ELGANs is easier to evaluate in the real world and may also be warranted because lung inflammation is very common below 28 weeks of gestation and because some neurodevelopmental benefits have been reported. In contrast, reducing potential harm should remain a priority. Therefore, further evidence is needed to answer whether the use of prophylactic hydrocortisone :

- should be restricted to infants born before 28, 27, or 26 weeks of gestation or younger, based on mortality/BPD risk, adjusted for center performance?
- may be limited to infants with severe respiratory distress syndrome requiring exogenous or hemodynamic failure?
- should be targeted to infants born after confirmed chorioamnionitis only?
- should include infants with IUGR even though they are more vulnerable to gastrointestinal complications?
- should be initiated immediately after birth or as late as up to 48 h?
- need to last 10 days or be discontinued earlier if the newborn is completely stable?
- can be used concomitantly with early PDA treatment with Ibuprofen?

Finally, there are still some obstacles to the implementation of prophylactic hydrocortisone, as knowledge in this area is evolving rapidly. Several guidelines are still cautious.^54,55^ because only one large RCT demonstrated the benefits of prophylactic hydrocortisone, and because long-term reassuring data were only recently published.^56^ Other meta-analyses and meta-regression even recently published unfortunately have looked at treatment effect and safety of different regimens or glucocorticoid molecules. Real world data on the most immature ELGANs under 24 weeks, not enrolled in previous RCTs, the combination between prophylactic and selective treatment strategies, and relevant and potentially parent-driven^57^ long-term outcomes not necessarily limited to BPD are also some of the challenges we should address in the near future.
